# Towards a Generic Residential Building Model for Heat–Health Warning Systems

**DOI:** 10.3390/ijerph182413050

**Published:** 2021-12-10

**Authors:** Jens Pfafferott, Sascha Rißmann, Guido Halbig, Franz Schröder, Sascha Saad

**Affiliations:** 1 Institute of Sustainable Energy Systems, Offenburg University of Applied Sciences, 77652 Offenburg, Germany; sascha.rissmann@hs-offenburg.de; 2 Deutscher Wetterdienst, 45133 Essen, Germany; Guido.Halbig@dwd.de; 3 Metrona Union GmbH, 81379 Munich, Germany; f.schroeder@metrona-union.de; 4 agl Hartz Saad Wendl Landschafts-, Stadt- und Raumplanung, 66111 Saarbrücken, Germany; saschasaad@agl-online.de

**Keywords:** heat–health warning system, building simulation, generic models, monitoring campaigns, statistical methods, indoor heat stress, thermal comfort, residential buildings

## Abstract

A strong heat load in buildings and cities during the summer is not a new phenomenon. However, prolonged heat waves and increasing urbanization are intensifying the heat island effect in our cities; hence, the heat exposure in residential buildings. The thermophysiological load in the interior and exterior environments can be reduced in the medium and long term, through urban planning and building physics measures. In the short term, an increasingly vulnerable population must be effectively informed of an impending heat wave. Building simulation models can be favorably used to evaluate indoor heat stress. This study presents a generic simulation model, developed from monitoring data in urban multi-unit residential buildings during a summer period and using statistical methods. The model determines both the average room temperature and its deviations and, thus, consists of three sub-models: cool, average, and warm building types. The simulation model is based on the same mathematical algorithm, whereas each building type is described by a specific data set, concerning its building physical parameters and user behavior, respectively. The generic building model may be used in urban climate analyses with many individual buildings distributed across the city or in heat–health warning systems, with different building and user types distributed across a region. An urban climate analysis (with weather data from a database) may evaluate local differences in urban and indoor climate, whereas heat–health warning systems (driven by a weather forecast) obtain additional information on indoor heat stress and its expected deviations.

## 1. Introduction

Heat–health warning systems use weather forecasts to predict heat stress. Most warning systems in operation are restricted to the forecast of outdoor heat stress. However, it is well known that the lack of night-time heat dissipation indoors is particularly problematic for health. Hence, a more practice-relevant warning system should forecast both the indoor and the outdoor heat stress [1].

In this study, indoor heat stress in passively cooled residential buildings is defined by the operative room temperature only. According to EN 16798 [2], the operative room temperature is the arithmetic mean value of the indoor air temperature and mean radiant temperature for an assumed air speed of 0.2 m/s. As the other impact variables on the perceived temperature (e.g., air humidity or air speed) are often uncertain, or even unknown, the calculation is simplified in the building simulation model, according to ISO 13790 [3], and does not consider the physiological heat balance, with regard to ISO 7730 [4]. In comparison to indoor heat stress, the outdoor heat stress is calculated with a physiological heat-balance model and results in the so-called UTCI universal thermal comfort index [5]. In Germany, a heat–health warning is defined by an exceedance of 32 °C for strong or 38 °C for extreme outdoor heat stress, respectively, during daytime, with 26 °C representing insufficient heat dissipation during nighttime [6].

As most people live in multi-unit residential buildings in cities, building models using in heat–health warning systems should describe those buildings as precisely as possible. Furthermore, the heat-stress forecast should particularly consider the urban climate. The urban heat island is a typical feature of the urban climate. Starting from the definition given by the World Meteorological Organization [7] it is characterized by the difference in air temperature between the hotter city and its cooler surrounding countryside; it reaches its maximum during nighttime under cloudless and calm weather conditions. The air temperature in cities depends strongly, in part, on building geometry, the thermal properties of the building fabric, radiation properties of the urban surfaces, and anthropogenic thermal release, e.g., domestic heating and cooling, traffic, and industry.

Heat–health warning systems and urban climate analyses face a similar challenge. While a heat–health warning system aims at forecasting heat stress as precisely as possible [8], an urban vulnerability analysis interprets the urban heat island effect around and inside buildings and may be used for urban planning, e.g., op. Furthermore, a warning system is driven by a weather forecast [9], while a climate analysis uses existing data from a weather database, e.g., according to guideline VDI 3787 [10]. Though both applications use different input variables, they both rely on building models.

In regionally or locally distributed ensemble simulations, the exact nature of the present building types and, in particular, of user behavior is not known. A generic building model can represent typical indoor conditions, without precise information on building parameters and user models [11].

Simulation models with very different characteristics, field studies on thermal comfort or heat stress in summer, and procedures for locally distributed simulation studies are well-known, e.g., EnergyPlus [12], ESP-r [13], or TRNSYS [14]. The results and findings have found their way into standards and widely-used applications.

However, no building models are available, which can easily be applied to ensemble simulations either for predicting a heat–health warning alert or evaluating the urban heat island effect and its impact on indoor heat stress.

In this study, measured room temperatures in typical, multi-unit residential buildings and measured weather data are used to develop a generic building model for simulation studies on thermal comfort in summer and heat stress. We apply a numerical building simulation model to the observations and use statistical methods for the evaluation of both the measured and the simulated data. The generic building model describes the thermal behavior of a typical, urban residential building. Since all data are from Germany, the model is valid for German cities only, but may be applied directly or with adaptions to other regions with moderate summer climate (i.e., temperate climate zone) but different building standards or user behaviors.

There are a large number of studies on thermal comfort and overheating in summer. As we want to develop a generic simulation model for residential buildings, based on a statistic analysis of measured room temperatures, we focus on studies with observations and sort out studies on non-residential buildings (e.g., office buildings, hospitals, schools, community buildings).

As typical residential buildings in temperate climate zones are not actively cooled and are manually controlled by the users in summer, we also skip lab studies (e.g., [15]) and studies on control strategies, air-conditioning, or other HVAC systems, specific technologies (e.g., green facades, cool roofs, urban, and micro-climate), and studies in hot and humid regions (e.g., Latin America, South Asia, or Africa).

A literature review shows both the state of the art and the research gap. Two international databases on observations are available: the ASHRAE 884 and SCATS databases.

The ASHRAE Research Project 884, on adaptive thermal comfort [16], required a large database of field observations around the world. They included thermal comfort questionnaire responses, plus coincident indoor and outdoor climatic observations. The ASHRAE Global Thermal Comfort Database II [17] provides monitoring data and is regularly extended by researchers from different institutes worldwide.

The SCATS database [18] contains thermal comfort field studies across Europe. These data have been used to develop a control algorithm. These and additional observations from field studies have been used to develop the adaptive thermal comfort model [19].

The findings from these field studies (and from lab studies) have been established in international standards (esp. ISO 7730, ASHRAE 55 [20], and EN 16798). However, these monitoring data do not include long-term observations and have not yet been used for the development of generic building models for residential buildings.

Unfortunately, only few raw data of the following literature are publicly available. However, the methodology (e.g., concerning statistics and characteristics), results (e.g., concerning heat waves), and conclusions (e.g., on building physical standards and user behavior) have been considered in data evaluation and model development.

Conventional theories of thermal comfort were set up based on steady-state laboratory experiments. As these experiments do not represent the real situation in buildings, especially not when focusing on residential buildings, reviews and adaptations have been considered to extract acceptable temperature ranges and comfort scales from field studies in residential buildings [21].

Andargie et al. [22] reviewed studies that investigated occupant comfort in multi-unit residential buildings, in relation to environmental and non-environmental variables. As the generic building model has been developed for urban residential buildings, the same (or similar) user and temperature patterns can be found in both studies.

Both Loga et al. [23] and Feist [24] summarized experiences and empirical knowledge in residential buildings and concluded that buildings with a high summer and winter heat protection standard (e.g., passive houses) face less heat stress than buildings with a poor standard.

Based on the feedback from passive house occupants, Grove-Smith and Bosenick [25] proved that passive houses are more resilient during periods of hot weather than conventionally built buildings. Ozarisoy and Elsharkawy [26] investigated the thermal performance of a prototype building, in order to assess overheating in summer during a long-term heatwave period.

Obviously, the room temperatures are lower in buildings with good (summer and winter) heat protection if residents use the sun-protection system and night ventilation, specifically for nighttime cooling. However, without such precautionary behavior, or with insufficient thermal insulation in summer, these buildings become heat traps. Schröder [27] confirmed the heat-trap effect in buildings with good thermal insulation but inadequate solar protection by measurements. Correspondingly, Dartevelle et al. [28] statistically analyzed the relationship between measured and perceived comfort and concluded that summer thermal discomfort is frequent in nZEB houses in Wallonia.

Becker and Paciuk [29] conducted a field study in 205 dwellings in summer (measurement of hygro-thermal conditions and documentation of occupant responses and behavior patterns) and documented significant differences between the computed predicted mean vote, using *Fanger*’s model (according to ISO 7730) and the reported thermal sensation.

Due to the urban heat island effect, the outdoor temperature at each monitored building differs from the measured weather data. Zinzi and Carnielo [30] investigated the impact of the urban environment on the energy and thermal response of residential buildings. Ambient air temperature and relative humidity were continuously measured in four neighborhoods in 2015 and 2016. The monitored neighborhoods are distinguished by: location in the urban area, construction materials for buildings and pavements, and geometry of the urban texture. Data were also measured at a non-urban station and used as undisturbed reference.

Tsinonis et al. [31] statistically analyzed the summer comfort condition in Athens, Greece. Sakkaa et al. [32] investigated the room and outdoor temperature in low-income houses during the summer. Following their findings, Pantavou et al. [33] demonstrated that the daily number of patients that were most likely affected by heat strongly correlates with high heat loads. Many other studies use mortality rates during heat waves for similar statistical evaluations, e.g., Canoui-Poitrine et al. [34]. These (and many similar) studies confirm the correlation between extreme summer conditions and indoor heat stress in residential buildings.

Rupp et al. [35] and Lamberti [36] evaluated existing approaches and methods to assess thermal comfort. Since users adapt to and, hence, actively influence the thermal environment, models should be developed for residential buildings.

Starting from the experiences documented in the literature, we developed a generic building model from the measured room temperatures in urban multi-unit residential buildings. The building model describes the energy balance and, hence, the room temperature in residential buildings without air-conditioning in moderate summer climate. The building model considers different building standards and user behavior, with regard to manual control.

In Section 2, a literature review of the influence of user behavior and building standards on the heat load in residential buildings is provided. From a methodological perspective (Section 2.1), we statistically analyze measured room temperatures and weather data, in order to develop a numerical model for residential buildings in moderate summer climates. The numerical algorithm of the building and user model is described in Section 2.2. We apply the monitoring data (Section 2.3 and Section 3) to this model and develop a building and user model for typical German multi-unit residential buildings (Section 4). This generic model calculates the hourly room temperature as a deterministic mean value with a stochastic deviation. Strictly speaking, this model cannot be validated, since all observations have been used for the model parametrization, and no additional observations are available for an independent validation study. However, an extensive plausibility analysis is shown in Section 5. In Section 6, the model is applied to three different scenarios. A vulnerability analysis, urban climate simulation, and implementation into a heat–health warning system, which show that this model gives additional information on expected room temperatures and, therefore, indoor heat exposure, since the generic building model calculates both the mean room temperature and its deviation. The main findings are summarized in Section 7, with a brief outlook for future applications and research.

## 2. Materials and Methods

Starting from a statistical analysis of measured room temperatures and weather data from field studies (i.e., solar radiation and ambient air temperature), we developed a numerical model for multi-unit residential buildings in moderate summer climates.

During the planning process, building simulation was used to evaluate the indoor climate, to minimize the energy demand or optimize the system technology. As an individual building concept is simulated, the building model is parameterized project-specifically. If certain parameters are not known, corresponding values are available from planning manuals or standards.

In studies for regional or urban effects, ensemble simulations with locally distributed buildings are used to evaluate (e.g., urban climate simulation) or to forecast (e.g., heat–health warning system) room temperatures. Different to the parametrization of an individual building, generic values (i.e., building physical parameters and typical user models) should be available for ensemble simulations. These parameters should be as realistic as possible and can be derived either from observations or from standards and guidelines (e.g., ASHRAE 55, DIN 4108 [37], EN 16798, or IWU 2018 [38]).

### 2.1. Statistical Analysis of Measured and Simulated Room Temperatures

In this study, we used a simulation model that was based on an analytical solution of Fourier’s thermal conduction equation. The simulation model calculates the measured room temperature in residential buildings as accurately as possible, cf. Section 4.3. The building physical parameters were defined according to the year of construction and its corresponding building quality. User models concerning attendance, window opening, or manual solar control are available from studies and standards, cf. Section 4.1. Starting from these parameters, a statistical analysis of both measured and simulated room temperatures was used for parameter identification, cf. Section 4.2.

**Figure 1:**
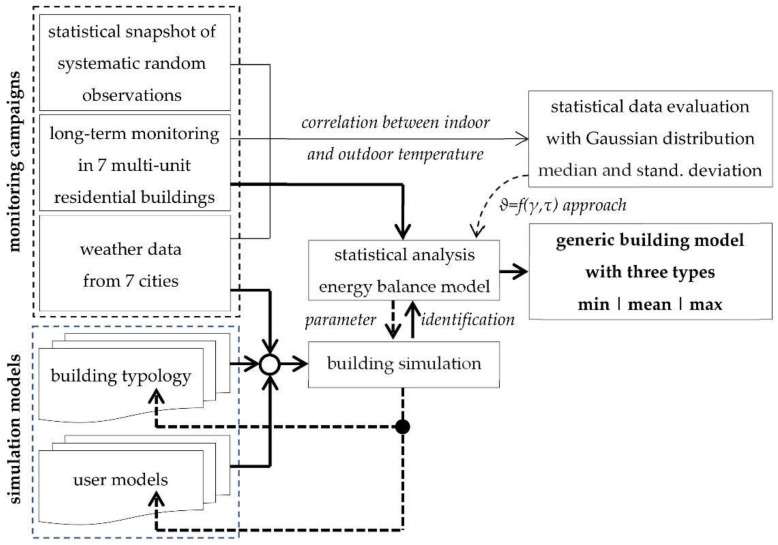
Methodology.

Figure 1 illustrates the methodology, which basically used statistical characteristics describing the stationary energy balance (for each day), the steady-state fluctuation (during a day), and the transient behavior (e.g., before and after a heat wave).

This novel approach is based on a statistical analysis of measured and simulated room temperatures only, whereas the building parameters are identified iteratively with a building simulation driven by solar radiation and ambient air temperature.

As the optimization criterion contains statistical values, the parameter identification necessarily leads to a parameter set with corresponding values, i.e., mean value and standard deviation. Hence, the simulated room temperatures do not need to match with the measured room temperatures in each time step, but they need to be true to the mean value of the measured room temperatures over a longer period. Furthermore, the building and user model should also describe the typical spread of room temperatures during the day, as well as the transient thermal behavior in summer.

### 2.2. Theory: Using a Resistance-Capacity Model for Model-Based Data Analysis

The simulation model, according to ISO 13790, was based on the analytical solution of Fourier’s thermal conduction equation:

(1) $$\rho \left(x,y,z\right)\cdot c\left(x,y,z\right)\cdot \frac{\partial T\left(x,y,z,t\right)}{\partial t}=\nabla \;\left[\lambda \left(x,y,z\right)\cdot \nabla \;T\left(x,y,z,t\right)\right]+q\left(x,y,z,t\right)$$

**Figure 2:**
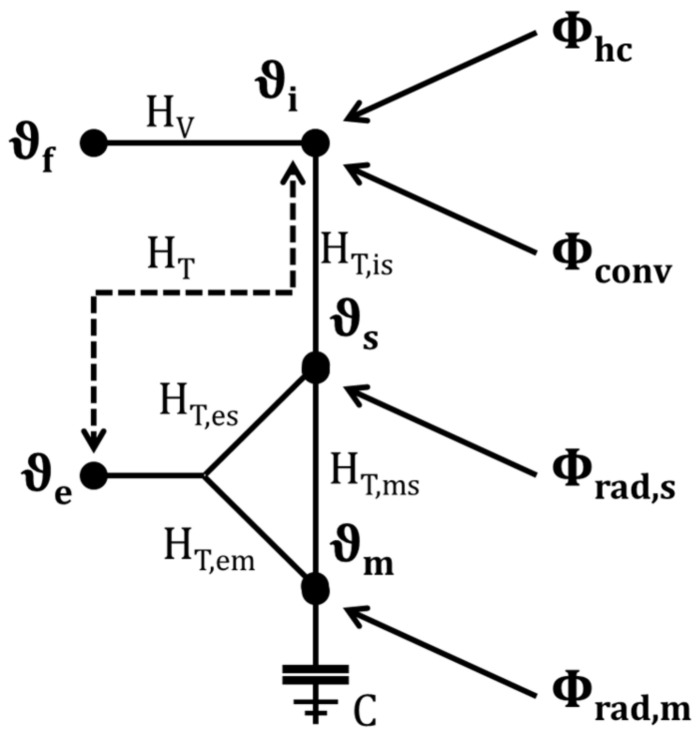
5R1C model, with five temperatures (ϑ) and four heat flows (ϕ), according to ISO 13790. Abbreviations and symbols: ϕ_hc_ (heat flow for heating and cooling), ϕ_conv_ (convective heat flow), ϕ_rad,s_ (radiative heat flow to the surfaces) and ϕ_rad,m_ (radiative heat flow to the thermal building mass; ϑ_f_ (air temperature close to the façade, ϑ_e_ (outside temperature) ϑ_i_ (room air temperature), ϑ_s_ (surface temperature) and ϑ_m_ (thermal mass temperature); H_V_ (heat transfer coefficient due to ventilation), H_T_ (heat transfer coefficient due to transmission), H_T,em_ (heat transfer coefficient due to transmission between ambient air and thermally heavy, opaque components), H_T,es_ (heat transfer coefficient due to transmission between ambient air and massless, transparent components), H_T,is_ (heat transfer coefficient room air and interior surface) and H_T,ms_ (heat transfer coefficient due to transmission between interior surface and building mass); C (storage capacity).

Considering location-independent material properties and constant heat transfer coefficients, the analytical solution results in a 5R1C model with five resistances (R) and a capacitance (C) [J K^−1^], according to Figure 2. The resistances are represented with the reciprocal value H [W K^−1^].

Heat flows (Φ) [W]: the internal and solar heat loads were divided according to their radiative or convective share in the heat flows (ϕ_conv_) to the room air (ϕ_rad,s_) to the surfaces and ϕ_rad,m_ to the thermal building mass. The convective heat flow (ϕ_hc_) for heating and cooling was set to zero in this study during the summer period.

Temperatures (ϑ) [°C]: the air flows into the building at an air temperature of ϑ_f_ close to the facade. The outside temperature (ϑ_e_) affects both the opaque (with thermal inertia) and the transparent (assumed to be massless) building components. The (mean) surface temperature (ϑ_s_) is linked to the room air temperature (ϑ_i_) and thermal mass temperature (ϑ_m_).

Storage capacity (C) [Wh K^−1^]: the thermal mass of the entire room is summarized in one storage capacity (C) (with the temperature ϑ_m_ of the thermal mass).

Heat transfer coefficient (H) [W K^−1^]: the heat transfer, due to ventilation (H_V_) was calculated for natural ventilation during summer with the driving temperature difference (ϑ_f_ − ϑ_i_). The heat flow due to transmission (H_T_) was calculated separately for the thermally heavy, opaque components (H_T,em_) and massless, transparent components (H_T,es_). Room air and surface are connected via H_T,is_ and surface and component via H_T,ms_.

ISO 13790 provides a complete set of equations for the simplified hourly method and the calculation algorithm.

The model has been extensively validated with high-resolution measurement data from an inner-city building, including records of user behavior and measurements of the urban climate close to the building [39].

### 2.3. Monitoring Campaigns

In this study, measurement data from three different data sources, from summer 2012, were used:

1. Room temperatures from 18 living rooms were recorded in seven buildings over several years in the project “Development of a reference indoor climate and a transient calculation method for the thermal assessment of buildings” [40]. The individual monitoring campaigns were carried out in accordance to ISO 7730 and scientifically evaluated. In the following, the recorded temperatures are interpreted as operative room temperatures and error-free. The seven buildings were located across Germany and also map three different summer climate regions in Germany.
2. Electronic heat cost allocators measure the temperature at the time of a manual reading for the plausibility check of the device quality. For the summer of 2012, 530,000 systematic random observations from a large number of buildings or apartments were available, thus providing an “anonymous, statistical snapshot of the summer room temperatures” [41]. In the present study, these measurements from the company *METRONA* were only evaluated if at least 12 individual measurements were available at the respective point in time. These (stacked) data were evaluated almost exclusively for the period Monday–Friday, between 8:00 a.m. and 7:00 p.m. Since neither the measuring method nor the placement of the sensors in the room met the requirements for comfort measurement, the data was interpreted accordingly, especially in comparison to the room temperatures recorded in detail in the *DFG* project. The measurements were roughly assigned to the maritime influenced north-west and continentally influenced south-east, in order to consider systematic differences, due to the prevailing climatic conditions.
3. Weather data (esp. outside temperatures and solar radiation) were provided by the German Weather Service [42]. With almost 90 [K d a-1] cooling degree days, the summer of 2012 was a typical summer. The weather data was recorded outside the cities and, consequently, did not consider the (local) urban climate in the neighborhood of the monitored buildings.

**Figure 3:**
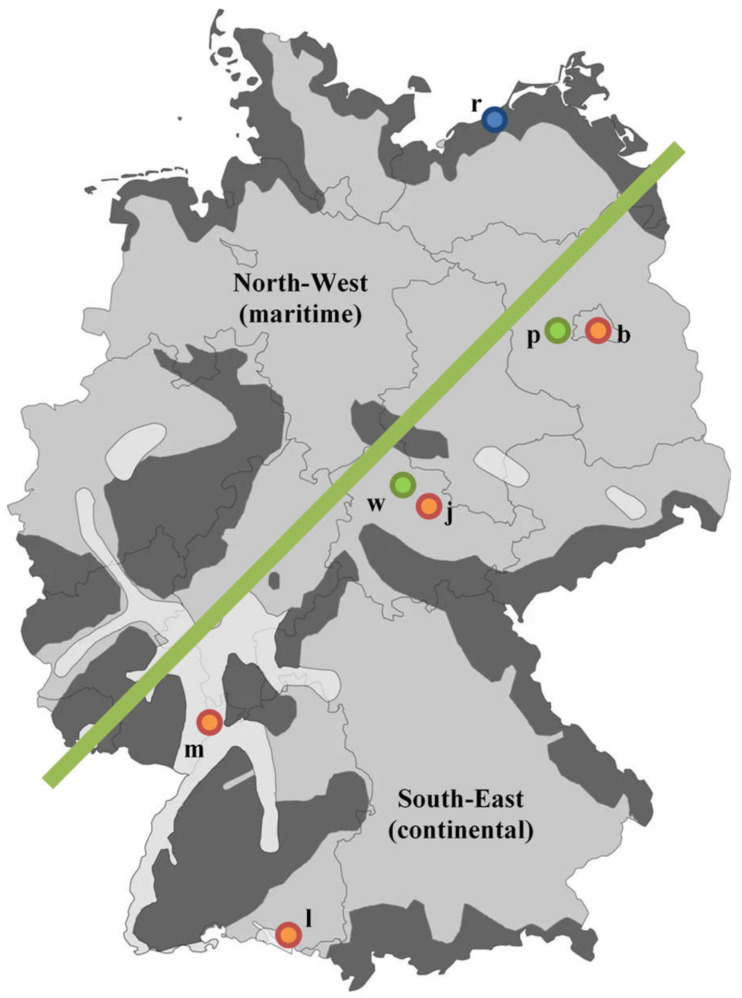
Measurement data from the summer of 2012. Seven cities in the *DFG* series of measurements in the three summer climate regions A, B, and C, according to DIN 4108-2: 2013-02 [37]. According to DIN 4108-2: 2003-07 [43], the locations are also marked as summer-cool (blue), moderate (green), and summer-hot (red), in accordance with the mean outside temperature in summer 2012. Each letter represents a site: r (Rostock), p (Potsdam), b (Berlin), w (Weimar), j (Jena), m (Mannheim) and l (Ludwigshafen). The mass data of the *METRONA* readings were roughly classified, according to the maritime/continental index. The so-called “continentality” indicates the extent to which the climate is shaped by land masses. In Germany, continentality increases from northwest to southeast.

Figure 3 shows the geographic allocation of the *DFG* and *METRONA* measurement data.

## 3. Analysis of Measured Room Temperatures

A time-dependent and statistical analysis of the monitoring campaigns in German residential buildings during the summer, which shows the fundamental correlations between outdoor and indoor temperature for different building types, building locations in the city, and user behavior.

### 3.1. Statistical Data Evaluation

Both the data of the *METRONA* readings and *DFG* monitoring campaign were evaluated hourly with:

- the minimum, mean, and maximum value;
- the standard deviation (68% of all values, between the 16% and the 84% quantiles);
- the 5% and 95% quantiles, as typical minimum and maximum values; and
- the 1% and 99% quantiles, to separate outliers.

**Figure 4:**
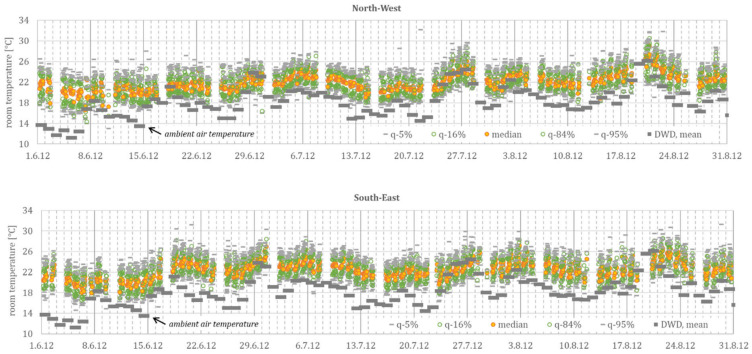
Monitoring data, summer 2012, from *METRONA* heat cost allocators. Only for Monday–Friday, between 8:00 a.m. and 7:00 p.m., during data reading.

Figure 4 shows the hourly sample, with 12 readings (minimum value) (and up to 1055 readings per hour), from the *METRONA* readings. The mean ambient air temperature from seven cities, representing different summer climate regions (cf. Figure 3), is additionally shown as reference.

**Figure 5:**
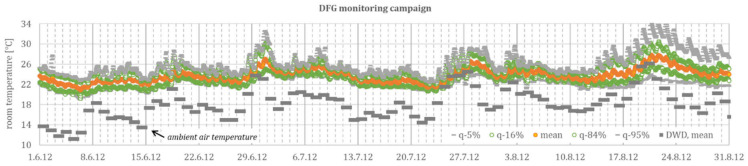
Monitoring data, summer 2012, from DFG monitoring campaign for 18 rooms in seven buildings.

Figure 5 shows the measured room temperatures from the *DFG* monitoring campaign and daily mean ambient air temperature.

It is noticeable in Table 1 that the *METRONA* readings (mean of all daily values ϑ_room_ = 22.2 °C for northwest and 22.6 °C for southeast) are consistently around 1.4 K below the *DFG* measurements (mean of all daily values ϑ_room_ = 23.8 °C). This is probably due to the non-representative positioning of the temperature measurement at the external wall, a low (long-wave) radiation exchange with the room enclosing surfaces, and the relatively low position in the room. Furthermore, the standard deviation for all measurements (3.1 K for the *METRONA* readings and 2.4 K for the *DFG* monitoring campaign), and especially during a heat wave (7.9 K for the *METRONA* readings and 5.1 K for the *DFG* monitoring campaign), is clearly higher than the *DFG* monitoring, which might be explained by the non-standardized position of the *METRONA* heat cost allocators.

Hence, the *METRONA* readings can only be used to a limited extent for an absolute evaluation but provide quantifiable information for statistical evaluation with scatter and dynamic progression. In this selected data set, distributed over different summer climatic regions and in buildings with different construction standards, the mean room temperature exceeds the mean ambient air temperature regularly by 5 K, and by 2 K during a heat wave, and the spread of measured room temperatures in a single room regularly reaches 5 K, with almost 8 K during a heat wave (with the upper and lower 5% quantile).

### 3.2. Room Temperatures during a Heat Wave

**Figure 6:**
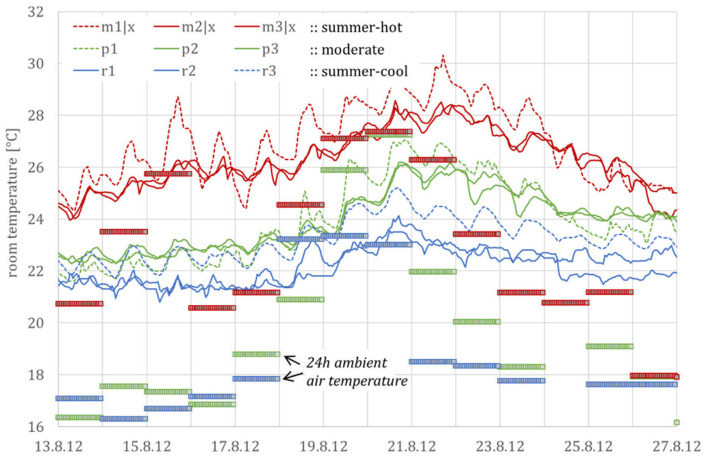
Measured room temperatures from the *DFG* monitoring campaign (three of seven locations) during the 14-day summer period 2012 from 13 to 26 August 2012. The measured values m1, m2, and m3, marked with |x, are presented again in the application example.

Figure 6 shows the temperatures from nine living rooms of the *DFG* monitoring campaign for a two-week period with summer days (with a maximum daily temperature of over 25 °C). The building m is in a hot-summer, building p in a moderate, and building r in a cool summer region.

The successive heating up and the slow cooling down can be clearly seen during and after the four-day heat wave (with daily maximum temperatures above 30 °C), from 18 to 21 August 2012. Additionally, night ventilation can be identified by the temperature gradient in only a few rooms and there, again, only in some nights.

### 3.3. Thermal Comfort Rating Versus Heat Stress

**Figure 7:**
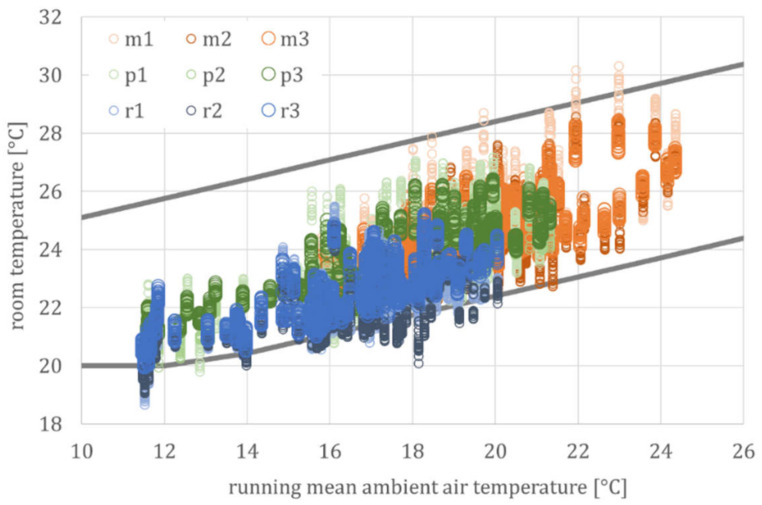
Class B comfort rating, according to EN 16798-1, for three selected buildings in summer 2012. The letters represent the sites Mannheim, Potsdam and Rostock according to Figure 3. The numbers represent three single rooms within each of the three selected buildings.

Figure 7 shows the comfort rating of these three *DFG* buildings. With an average summer temperature of 19.6 °C for the hot-summer region, 17.4 °C for the moderate, and 16.4 °C for the cool-summer region; the three buildings represent the three German summer climatic regions (cool, moderate, and warm). A good room climate of comfort class B, according to EN 16798-1, is achieved in all three buildings.

In building m, however, the minimum nighttime temperatures are over 26 °C on five days. This results in a severe indoor heat stress, despite the good comfort rating. Thus, heat stress should be detected independently from a thermal comfort assessment. 

## 4. Model Development

The simulation model, according to ISO 13790, was used for the model-based data evaluation of the observations. Following this data evaluation, a parameter identification resulted in a generic building model, which represents both a typical multi-unit residential building and its deviations, in terms of building physics and user behavior.

### 4.1. Definition of Building Parameters and User Models

For scaling purpose, a generic building model should be based on size-related data. Therefore, all building physical parameters are related to the external surface, since the surrounding surface is the model boundary. A_facade_ is the sum of all opaque and transparent external components A_i_:

(2) $$A_{\mathrm{facade}}\left[m^{2}\right]=\sum A_{i}$$

The energy balance can be reduced to a few main characteristics: heat losses, heat gains, and storage capacity.

The (specific) heat loss coefficient (H_T_) is determined with the area (A) [m^2^] and the heat transfer coefficient (U) [W m^−2^ K^−1^] for all (transparent and opaque) external components:

(3) $$H_{T}\left[\frac{W}{m^{2}K}\right]=\frac{\sum U_{i}\cdot A_{i}}{A_{\mathrm{facade}}}$$

The (specific) heat loss coefficient (H_V_), due to ventilation, is determined with the air exchange rate (ACH) in a room, the volume (V), and the specific heat capacity, ρcair = 0.34 Wh m^−3^ K^−1^):

(4) $$H_{V}\left[\frac{W}{m^{2}K}\right]=\frac{\sum {\mathrm{ACH}}_{i}\left[h^{-1}]\cdot V_{i}[m^{3}\right]\cdot {\rho c}_{\mathrm{air}}}{A_{\mathrm{facade}}}$$

The air exchange rate (ACH) varies between 0.1 and 3 h^−1^, depending on user behavior and, thus, deviates from the assumptions in DIN 4108-2. Schröder et al. [44] presented a user model for opening windows (o_win_) in residential buildings, based on a monitoring campaign in six buildings with 48 apartments. These results were compared to the method of Herkel et al. [45], with a daily profile and the likelihood model [46].

Derived from these four user models, the user behavior, with regard to manual window opening, follows a deterministic behavior, whereas the model itself is based on a statistical analysis [47]. Considering the temperature- and wind-induced pressure differences from wide-ranging monitoring campaigns [48], the individual user behavior for all buildings results in a time- and temperature-dependent model for the air exchange rate (ACH). Based on this model for manual window control, the parameter identification results in stepwise air change rates:

(5) $$\mathrm{ACH}\left[h^{-1}\right]=\left\{\begin{array}{l} \;{\mathrm{ACH}}_{\min},\;\;\;\;\;\;during\;the\;day\;\left(08:00-18:00\right)\\ {\mathrm{ACH}}_{\max}\cdot o_{\mathrm{win}},\;\;\;\;\;\;during\;the\;night\;und\;\vartheta_{i}>\vartheta_{e}\; \end{array}\right.$$

with

(6) $$o_{\mathrm{win}}=\left\{\begin{array}{c} 0.5,\;|\;\vartheta_{i}>22\;{}^{\circ}C\\ \;\;1,\;|\;\vartheta_{i}>24\;{}^{\circ}C \end{array}\right.$$

The energy balance shifts, due to the different solar and internal heat inputs. As a result, the actual air exchange in the three generic building models differ significantly from one another, despite of the supposedly identical user models. This corresponds with the typical user model for manual control of windows, which distinguish between a frequent and infrequent window opener.

The solar heat gain coefficient (S) is determined by the solar heat gain coefficient (g_tot_[-]) for all transparent external components (A_win_[m^2^]):

(7) $$S\left[\frac{m2}{m2}\right]=\frac{\sum g_{\mathrm{tot},i}\cdot A_{\mathrm{win},i}}{A_{\mathrm{facade}}}$$

with

(8) $$g_{\mathrm{tot},i}=g_{⊥,i}\cdot F_{C,i}\cdot F_{w,i}\cdot F_{f,i}\cdot f_{s,i}$$

according to DIN 4108-2.

g_⊥_ is the surface-normal solar heat gain coefficient of the glazing, F_C_ the reduction factor for the sun protection, F_w_ for soiling, and F_f_ for the frame portion. Depending on the simulation model, a further correction factor (f_s_) may consider the angle-dependent or non-surface-normal irradiation, static shading, or other aspects. Regardless of the user model, the solar protection (e.g., Venetian blinds or curtains) is closed, in the event of direct radiation on the transparent surface (from 200 W m^−2^). If the room has outer walls in different orientations, S must be determined (and simulated) separately for the individual orientations. Typically, user models distinguish between “Mr. Bright” and “Mr. Dark” for the manual control of shading devices. This is considered by the reduction factor F_C_.

Internal heat gains (q_intern_) depend strongly on the attendance and equipment. Typically, the user models consider single, family, and senior households.

The heat storage capacity (C) for all three building types corresponds to a thermally heavy building (with an effective storage capacity of 130 Wh m^−2^_nfa_ K^−1^) and is calculated for a typical room geometry, with Λ = 4.5, where Λ indicates the area ratio of all space-enclosing areas to the base area. The heat storage capacity (C) can be recalculated for heavier, and especially lighter, buildings or rooms with strongly deviating geometry, if Λ is adjusted accordingly or the effective storage capacity, based on whether the net floor area differs from 130 Wh m^−2^_nfa_ K^−1^, respectively.

Simulation models for parameter identification. Based on the German building typology [38] for the structural and physical parameters and user models, the simulation model contains a total of 1350 scenarios: five building types with three characteristics each, three presence profiles with three characteristics each, five user models for the ventilation behavior, and two models for the operation of the sun protection. The building-physical and user-related parameters are varied within practically relevant limits.

### 4.2. Parameter Identification

**Figure 8:**
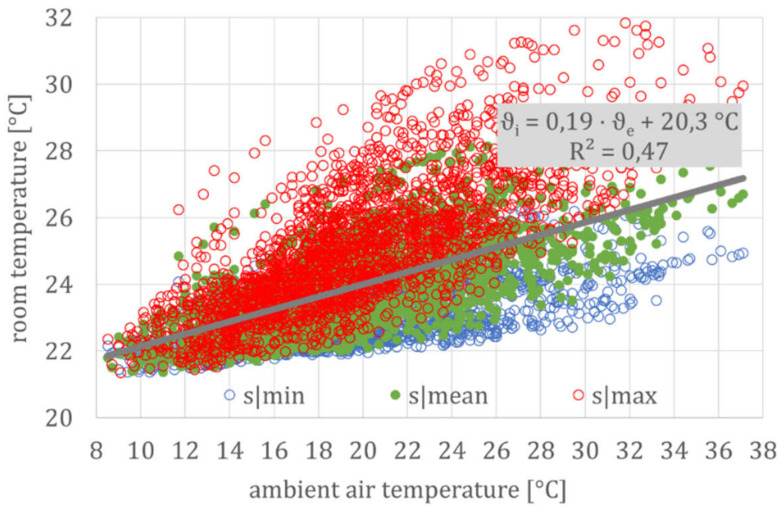
Simulation results for the three generic building models (according to Table 2) for the summer period. Abbreviations and symbols: s|min, s|mean, and s|max (simulation results from the three generic building models), ϑ_i_ (operative room temperature), ϑ_e_ (ambient air temperature) and R^2^ (regression coefficient).

The parameters are not identified separately for each region and building or even single rooms, but by the unified relation between indoor and outdoor temperature. Accordingly, the simulated and measured room temperatures are not compared directly but by their mean values and the corresponding standard deviations. Graphically, this corresponds to rising point clouds, each of which is described via a linear relationship between room and outside temperature and a scatter, cf. Figure 7 for the standardized evaluation of monitoring and Figure 8 for simulation results.

Table 2 shows the parameter set for three typical residential buildings in Germany, which could be determined with the help of a structured parameter identification. According to the user behavior, and depending on the weather situation, the internal and solar heat gains, and especially the air exchange (and thus H_V_ for ventilation), vary from day-to-day and from building to building.

If a mean value of room temperature and its daily course is calculated for a simulation period, the (mean) excess temperature γ [K] and time constant τ [h] for dynamic processes can be determined as characteristic values:

(9) $$\gamma \left[K\right]=\frac{\bar{Q_{\mathrm{solar}}}\left[W\right]+\bar{Q_{\mathrm{intern}}\left[W\right]}}{H_{T}\left[W/K\right]+\bar{H_{V}\left[W/K\right]}}$$

(10) $$\tau \left[h\right]=\frac{C\left[\mathrm{Wh}/K\right]}{H_{T}\left[W/K\right]+\bar{H_{V}\left[W/K\right]}}$$

These calculated values are between 3.5 and 5.4 K for the static excess temperature (γ) and between 60 and 100 h for the time constant (τ). Due to the cumulative effects, especially during heat waves, this provides a large spread between cool, normal, and warm residential buildings of over 5 K, which strongly corresponds to the spread of the measured room temperatures, documented in Figure 4 and Figure 5, respectively.

Three generic building models were based on a Gaussian distribution of typical building and user models. All parameters were determined from measured values using statistical methods. The parameter identification was based on two objective functions, with regard to the excess temperature (γ) and the time constant (τ). The parameter sets were reduced to five characteristic values: H_T_, S, q_intern_, ACH, and C. These values describe the energy balance of generic building types and, hence, independently from a specific building. The identified parameters are valid for typical multi-unit residential buildings in German cities.

### 4.3. Reliability of Generic Building Parameter

Figure 8 shows the temperature signatures of the simulation run with the three generic building models. This graph shows the typical point cloud (represented here by the regression line and coefficient of determination for the mean building model), based on hourly values.

**Figure 9:**
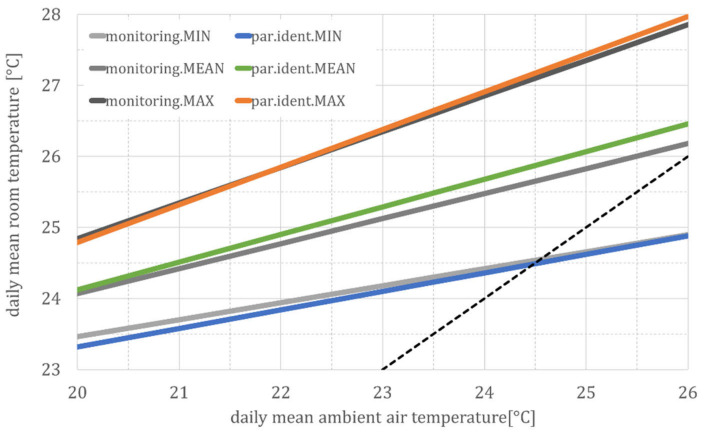
Daily mean values of the measured and simulated room temperatures, parameterization according to Table 2. Abbreviations and symbols: MIN, MEAN and MAX (according to the three generic building models), monitoring (regression line for measurement data) and par.ident (regression line according to the parameter identification).

If the complex relationship between room and outside temperature is reduced to a linear regression, the thermal building behavior can be directly compared. Figure 9 shows a comparison of the daily mean of all measurements from the *DFG* project with these three simulation models.

For clarity, only the regression lines for the mean temperatures for the three types of a cool (MIN), normal (MEAN), and warm (MAX) building are shown here. Since the individual relationships are linear, or at least partially linear, the superposition principle can be applied. This leads to an approximate linear building model with a corresponding deviation, whereas the deviation can be explained by the time delay and temperature damping, due to the thermal inertia of the building, since both the time delay and temperature damping were not considered in this time-independent correlation between room and outside temperature.

**Figure 10:**
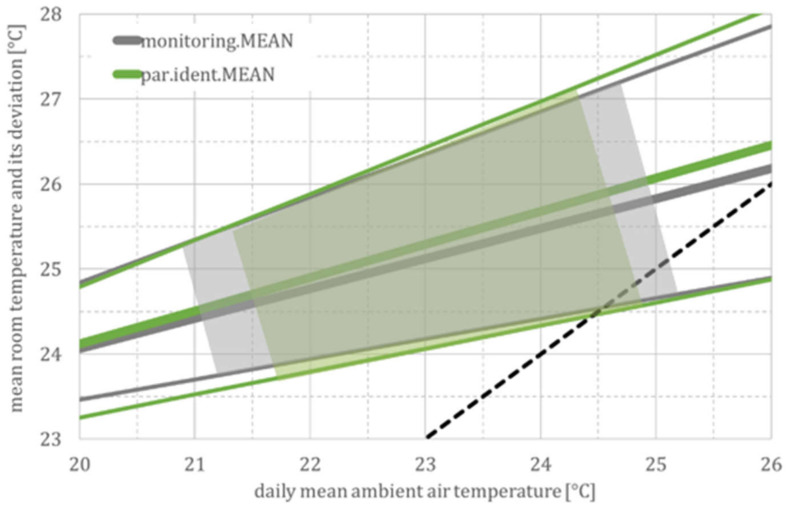
Room temperatures and their daily fluctuations for the MEAN building model. Abbreviations and symbols: monitoring (regression line for measurement data) and par.ident (regression line according to the parameter identification).

In addition to the regression line for the mean values of each building model, Figure 10 exemplarily shows the regression line for the daily fluctuation for the MEAN building type. As expected, the daily fluctuations in measurement and simulation largely coincide and increase from ±1.5 K at moderate to ±3 K at high summer temperatures. Due to the higher night-air exchange at high outside temperatures (see user model, especially Equation (6)), the coupling factor H_T_ + H_V_ increases and, as a result, the time constant (τ) decreases. This leads to less damping and, thus, to greater fluctuations.

Considering these daily fluctuations for the cool and warm buildings, the spread of all simulated values (ϑ_par_._ident_) at high ambient air temperatures is between just under 24 °C (lowest daily temperature in a cool building) and over 29 °C (highest daily temperature in a warm building). Again, this corresponds to the spread of the measured values (ϑ_monitoring_) shown in Figure 5 and Table 1 for the *DFG* monitoring campaign.

This demonstrates clearly that the stepwise data compression, using statistical methods and the 5R1C-thermal network, results in a robust building model with a suitable thermal inertia. From a methodological perspective, these generic models can be (moderately) extrapolated beyond the range of values used, as none of the parameters were restricted, with regard to the building or user model and weather data.

## 5. Model Validation

As all measured data have been used for the model parametrization, there is no independent data set available for a model validation available. Thus, an extensive plausibility check, based on two recursive simulations, proves the method.

Indeed, generic models can be easily applied in simulation studies. However, for methodological reasons, these models have lost their direct applicability. Two practical examples show how the generic models can be applied in urban climate evaluations and heat–health warning systems. For consistency in this study, building m from the *DFG* monitoring campaign (cf. Figure 5) is used as an example again. Furthermore, the measured *DWD* weather data are applied to these simulations.

### 5.1. Evaluation of the Room Temperature in One Building

**Figure 11:**
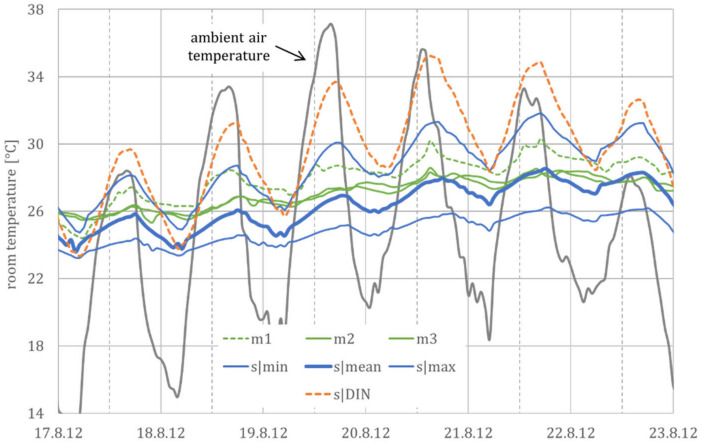
Measured and simulated room temperatures for a four-day hot spell in August 2012. The days before (17 August 2012) and after (22 August 2012) the hot spell are also shown for the evaluation of the transient upward and downward trend. Abbreviations and symbols: m1, m2 and m3 (measured room temperature in three rooms at the Mannheim site), s|min, s|mean and s|max (simulation results from the three generic building models) and, for comparison, s|DIN (simulation results from the building model using the user profiles from DIN 4108-2).

The first example is to estimate the room temperature for the residential building m (in the hot-summer region). Figure 11 shows that the measured room temperatures m2 and m3 correspond with a mean building behavior, as well as m1 with a warm building. As expected, the generic model shows a slightly larger temperature fluctuation during the day.

Additionally, this figure clearly shows that the assumptions from the standardization (application of the user models from DIN 4108-2 to the generic MEAN model) result in a worst-case scenario and are, therefore, hardly suitable for a realistic estimation of the indoor climate.

As the generic model uses generalized assumptions, concerning the building standard and the user behavior, it does not aim at a precise calculation of the current room temperature but at an approximate forecast of the mean room temperature and the accompanying (daily) fluctuation. In this sense, the estimated deviation provides additional information on feasible minimum or maximum room temperatures.

**Figure 12:**
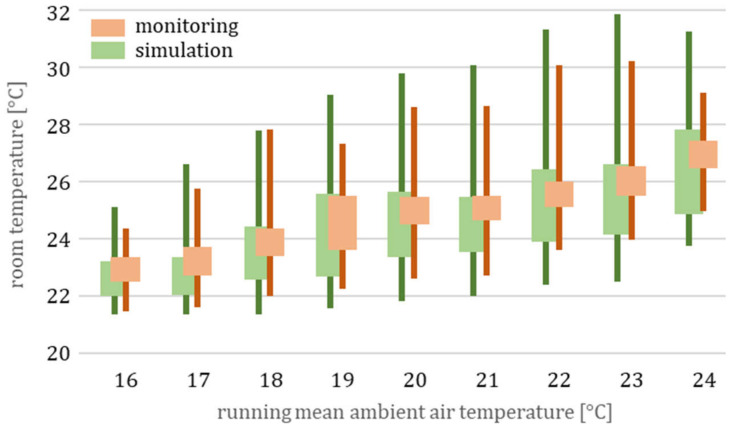
Monitoring and simulation: temperature signatures with minimum, standard deviation, and maximum for the period from 1 June to 31 August 2012, cf. comfort assessment for the building m in Figure 6.

Figure 12 shows a precise forecast of room temperatures (and heat stress). All monitoring and simulation data are summarized in 1 K temperature sections for the running mean temperature, according to EN 16798.

To conclude, the generic model is able to estimate the minimum, average, and maximum room temperatures for this residential building, according to Table 2, though the actual building description is unknown.

### 5.2. Evaluation of Expected Room Temperatures in a Building Ensemble

The second example is to evaluate the urban climate for the hot-summer region. Again, building m is used as an example. For this purpose, a frequency distribution or, alternatively, the evaluation of overheating hours [h a^−1^] or overheating degree hours [Kh a^−1^] of the expected room temperatures can be used for a vulnerability analysis.

**Figure 13:**
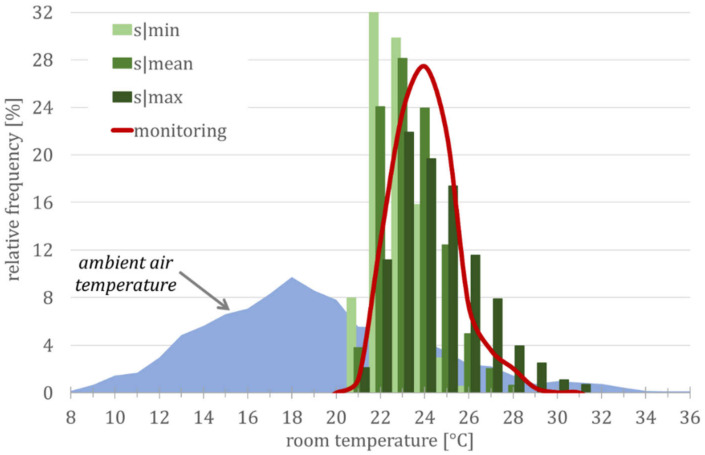
Frequency distribution of room temperatures in the period from 1 June to 31 August 2012. Abbreviations and symbols: s|min, s|mean and s|max (simulation results from the three generic building models).

Figure 13 shows the temperature behavior in a typical urban district consisting of buildings with different construction standards and different user profiles. As expected from Figure 5, the mean and the maximum generic models show a similar frequency distribution of temperatures as the observations.

Table 3 gives some quantifiable heat stress indicators. The simulated overheating and overheating degree hours are within the range of the measured values.

## 6. Application Scenarios

The impact of the building standard (esp. with regard to winter and summer heat protection) and user behavior (esp. with regard to the use of shading devices and window control) on indoor heat stress is controversially discussed in literature (cf. Section 1.3) and day-to-day practice. Hence, a sensitivity analysis was carried out for different building standards and user models by Pfafferott et al. [49], in order to develop a building model for the German heat–health warning system:

- The highest room temperatures are reached in attic apartments with poor (summer and winter) thermal insulation.
- Moderate room temperatures prevail in typical residential buildings.
- The lowest room temperatures are expected in buildings with a good thermal insulation and external shading devices.
- In each concept, the user behavior, with regard to window opening, has a strong effect on the room temperatures [50].

A building simulation model was identified based on a statistical analysis of all simulation results. Additional to the so-called warning scenario (with a heavy-weight building type and appropriate user behavior, according to the median room temperature of all buildings), a so-called extreme scenario (with a light-weight building type and improper user behavior according to the warm 84% percentile) has been defined for the numerical forecast of indoor heat stress.

Against that, the generic building model provides three building types that have been derived from observations and a model-based data evaluation. This model can be favorably used for ensemble simulations in complex scenarios.

### 6.1. Vulnerability Analysis

Starting from an urban climate analysis based on monitoring data (e.g., measurement runs or local temperature stratification and wind speed), the weather data can be taken from test reference years [51], with regard to different city areas, such as the city center, urban/suburban area, or surroundings.

The building simulation models are then applied, with regard to the building structure, e.g., historic buildings in the city center, buildings from the 1950s and 1970s in the urban area, buildings from 1990s, and new passive houses in the suburban area. Each of these building types can be described with the generic building model (on the basis of Table 2).

**Figure 14:**
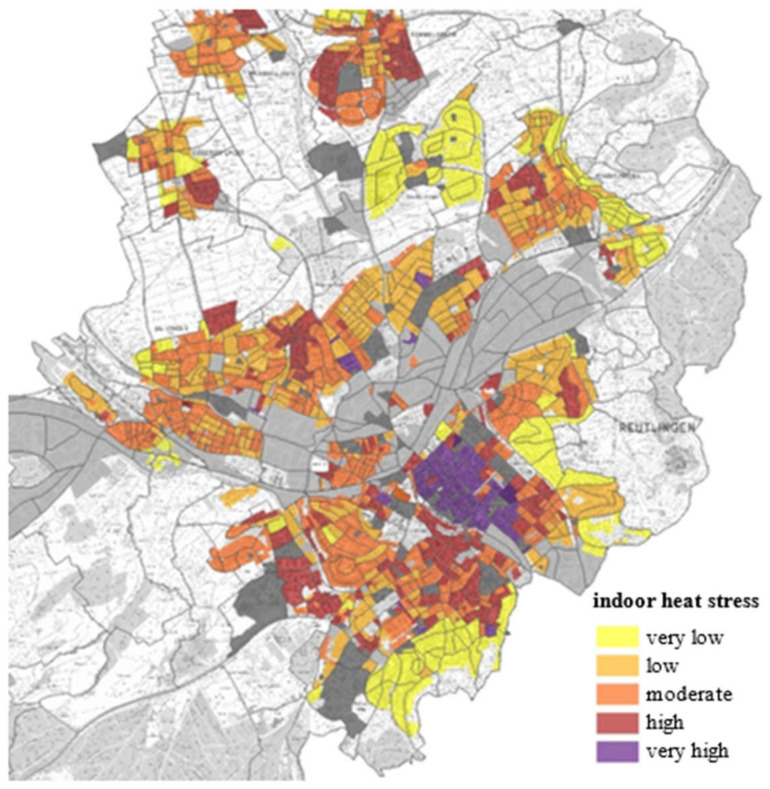
Vulnerability analysis for Reutlingen, excerpt from an urban heat stress map, cited by [52].

Considering both the percentage of each building standard in a certain district and its frequency distribution of operative room temperatures, the indoor heat stress can be evaluated for each city district [52]. Figure 14 shows, exemplarily, an indoor heat stress map from a vulnerability analysis for Reutlingen, Germany.

### 6.2. Urban Climate Simulation

**Figure 15:**
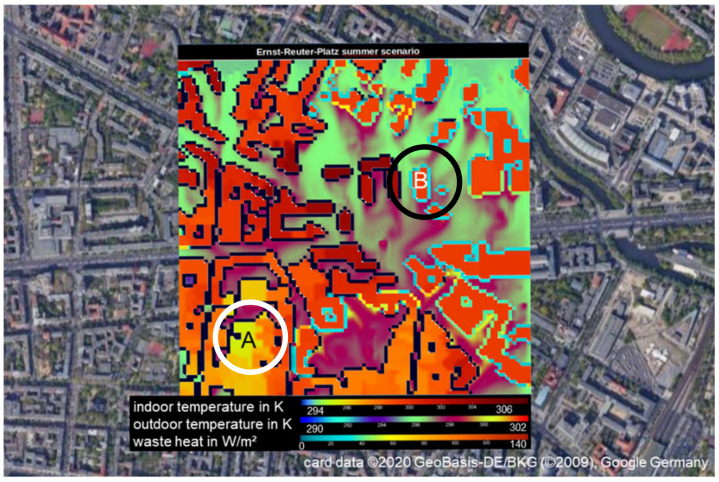
Excerpt from an urban climate simulation. The Ernst-Reuter-Platz in Berlin connects five urban street canyons and is surrounded by high-rise buildings. The simulation runs with a resolution of 1 m × 1 m × 1 m. The outdoor temperature is around 24 °C in the summer scenario. The graph shows the indoor and temperature, and the anthropogenic waste heat at the outside surface, depending on the air conditioning concept. Façade elements with no anthropogenic waste heat (i.e., buildings with passive cooling in summer) are shown in black. From a PALM simulation, run by Leibniz University Hannover, cited by [53].

Urban climate simulations focus on the heat stress in the city. Both the city structure (e.g., street canyons, open places, vegetation, or water) and density of residential and non-residential buildings have a strong impact on the indoor and outdoor heat stress, since there is a strong interaction between the urban atmospheric canopy layer and building energy balance. Figure 15 shows a snapshot result from a simulation run for Berlin with the PALM model system [11].

Due to night ventilation and low heat gain, the room temperature of 23 °C in the residential building A (white circle) is, at this moment, lower than in the office building B (black circle), which is air-conditioned for a room temperature of 26 °C. Accordingly, the cooling energy (ϕ_hc_) (in Figure 2) is unequal from zero in building B. This results in a positive waste heat from air-conditioning systems.

The generic building model is suitable for ensemble simulations if the building parameters (and the user behavior) are uncertain or even unknown.

### 6.3. Heat–Health Warning System

Koppe [54] describes the German heat–health warning system, which has been run by the German Meteorological Service *DWD* since 2006 and was extended with a prediction of the indoor heat stress in 2011. A heat–health warning will be published by the meteorologist on duty (for today), if both the outdoor and indoor heat stress may exceed a harmful-to-health limit for several days. The outdoor heat stress is identified by the perceived outdoor temperature UTCI [5] and indoor heat stress by the operative room temperature [39].

The German heat–health warning system [55] considers both the maximum outdoor heat stress during daytime and minimum indoor heat stress during nighttime, in which the perceived outdoor temperature UTCI and operative room temperature are used to calculate the thermal stress. If the UTCI exceeds 38 °C outside during the day and operative room temperature exceeds 26 °C inside during the night, the heat stress is rated as “extreme”.

**Figure 16:**
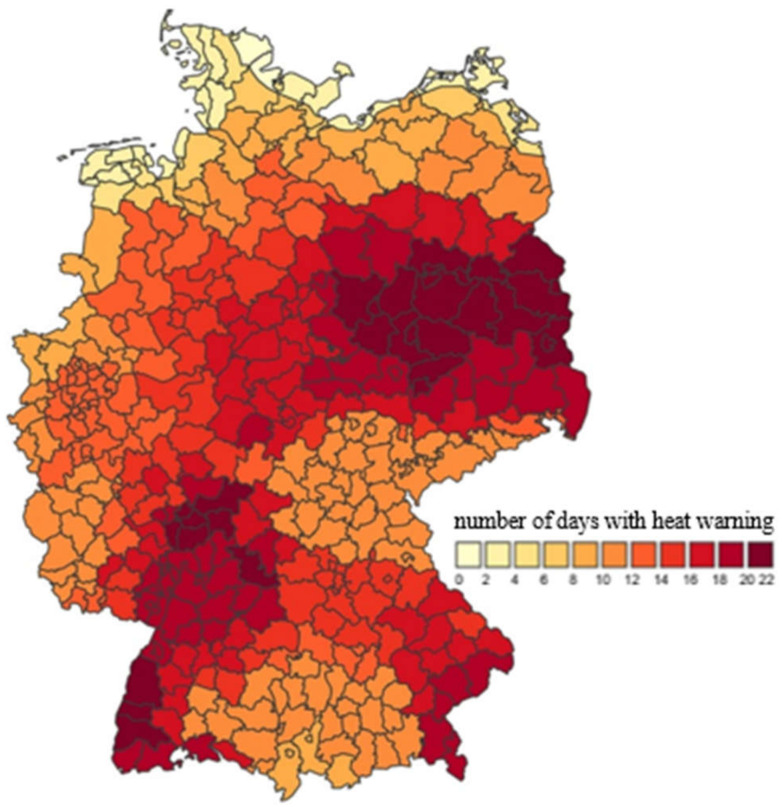
German heat health warning system: number of days with a heat warning in each district in 2018; source: German Meteorological Service (DWD).

Building simulations for 4500 locations in Germany forecast the heat stress in 294 districts for the next days. Figure 16 shows, exemplarily, the announced heat warning days in the summer of 2018 for 294 German districts. Warnings of very strong and extreme heat exposure were issued in individual counties on up to 22 days.

Since the heat-heath warning system considers both the weather forecast and simulated room temperatures, generic building models can be used to forecast the room temperatures as realistically as possible.

**Figure 17:**
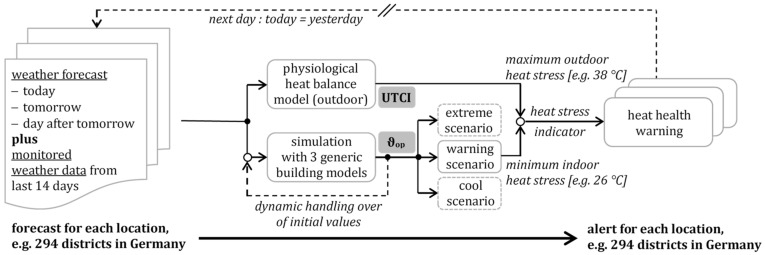
Algorithm for the integration of simulation with generic building models in a heat health warning system. The outdoor and indoor simulation models were run only by measured and forecasted weather data: ambient air temperature (ϑ_e_), relative air humidity (φ), wind speed (v), and solar radiation (G_s_).

Figure 17 represents an algorithm, which is basically developed on the *DWD* algorithm with the evaluation of outdoor [54] and the indoor [49] heat stress. The proposed new algorithm applies generic models to the building simulation, in order to:

- issue a heat health alert using the mean building type;
- give additional information on the probability of harmful overheating in specific buildings (e.g., in city center, in upper apartments or even on top floor, with high solar exposure or with poor building standards), using the maximum building type; and
- estimate lower indoor heat stress (e.g., enhanced night ventilation, better solar protection, or cooler rooms in the apartment) using the minimum building type.

The heat–health warning algorithm works with the weather data as input only. The forecast is implemented into a shifting time loop. Each day starts with the measured weather data from the past and uses the weather forecast for the next days. Accordingly, the last day of observations is dropped out. 

The outdoor heat stress is evaluated based on the perceived temperature UTCI [°C], with indoor heat stress evaluated based on a building simulation with three generic building models.

Although the statistical analysis focused on the correlation between the measured room temperature and corresponding and ambient air temperatures, it should be repeated that the building model was based on a 5R1C-model, shown in Figure 2. This model used the ambient air temperature and solar radiation from the weather data. Neither the wind speed and direction nor the air humidity were used in this model, since the impact of wind on the air volume flow and of the air humidity on the physiological heat balance highly depends on the specific building and its location. Both effects cannot be directly described by a generic model. Worth mentioning, the weather data can be applied to the model considering the urban heat island effect for urban areas. The driving weather data determine the heat flow by transmission (represented by H_T_) and ventilation (represented by H_V_), as well as the solar heat gain ϕ_rad_ in the building model, cf. Section 2.2.

## 7. Conclusive Remarks

Room temperatures in manually controlled residential buildings are stochastically distributed, rather than deterministically described. A generic building model with statistically justified parameters considers the thermal behavior of a typical building and its deviations. As the parameter set is reduced to a few main characteristics, the presented model can easily be applied to ensemble simulations with many different buildings.

Ensemble simulations are used in heat–health warning systems (for a large region) to predict current heat stress and, in urban climate analyses (for a city or a city district), to evaluate heat stress. In both applications, the accuracy strongly depends on the reliability of the parameter set, which is applied to the simulation model.

A wide-range data set of measured room temperatures from different monitoring campaigns was statistically evaluated to define building characteristics for the calculation of room temperatures in summer.

We applied both statistical methods and a model-based data evaluation to a comprehensive and consistent set of measured room temperatures and weather data. In addition to scatter plots and (sectional) linear regression models, frequency distributions were also used for parameter identification. Based on this approach, we developed a generic building model, which consists of three building types. This generic model represents a multi-unit residential building with a mean building standard (for German cities) and a typical user behavior. Considering the standard deviation from the statistical data analysis, two additional building types represent a building, which provides a cooler or warmer environment, respectively. Here, it is irrelevant how the individual parameters relate to each other; it is only important that a realistic situation is represented by the parameter set.

As all observations were used for parameter identification, the building model could not be strictly validated with an independent data set. However, the building model was applied to each data set separately. Consequently, this recursive application of a single data set to a model which is based on a multiple data set results in a plausibility check but not in a mathematically complete validation.

The generic building model was exemplarily applied to a vulnerability analysis, considering heat stress (analytical simulation model for a long-term simulation for a summer period), and urban climate simulation (numerical simulation model for a short-term simulation for one summer day). In both scenarios, the room temperatures in many buildings were calculated as a function of the prevailing weather data and urban heat island effect. The generic building model compensates for unknown building characteristics, since the building standard for each building in both simulation scenarios could be identified by its year of construction only.

Heat–health warning systems should use both the maximum outdoor heat stress (during the day) and minimum indoor heat stress (at night) as a significant and appropriate heat stress indicator. The indoor heat stress can be favorably estimated with the operative room temperature from a building simulation, which calculates the expected mean room temperature and its (standard) deviation.

The consideration of indoor heat stress in residential buildings bridges the gap between a heat forecast and an efficient heat–health warning system with valuable information on extreme situations, based on the expected mean room temperature. Furthermore, the stochastical distribution of feasible room temperatures shows the effect of appropriate user behavior to counteract heat stress. The information on heat stress and counteracting measures may be interpreted differently for the publicity and health-care services [56].

Generic building models can be enhanced with more consistent data from long-term monitoring campaigns and field studies. A broader application of generic building models in simulation studies yields both expert and empirical knowledge. These experiences may lead to improved and extended data sets for future applications in heat–health warning systems, to alert about an approaching heat wave, and in urban climate analyses, to find measures how to protect our cities from heat stress.

## Figures and Tables

**Table 1:** Statistical evaluation of mean values and standard deviations.

	*METRONA* Readings (Mon–Fri 07:00–19:00)	*DFG* Monitoring
room temperature (summer period)	22.4 °C ± 3.1 K	23.8 °C ± 2.4 K
room temperature (heat wave)	24.3 °C ± 7.9 K	26.2 °C ± 5.1 K
ambient air temperature (summer period)	18.1 °C ± 2.7 K	
ambient air temperature (heat wave)	24.3 °C ± 3.8 K	

**Table 2:** Parameter identification for three generic building and user models, based on statistical methods.

	par.ident Min	par.ident Mean	par.ident Max	
H_T_	0.19	0.43	0.87	W m^−2^_facade_ K^−1^
ACH_min_/ACH_max_	0.1/3.0 ^(4)^	0.1/3.0 ^(4)^	0.1/3.0 ^(4)^	h^−1^
S	0.036	0.067	0.159	m^−2^_facade_
q_intern_	100 ^(1)^	220 ^(2)^	260 ^(3)^	Wh m^−2^_facade_ d^−1^
C	186	186	186	Wh m^−2^_facade_ K^−1^
for information only, based on simulation for summer 2012				
γ	3.5	4.3	5.4	K
τ	96	76	61	h

^(1)^ 4 W m^−2^_nfa_ from 18:00 to 08:00 (typical single household); ^(2)^ 6 W m^−2^_nfa_ from 18:00 to 08:00, 2 W/m^2^_NGF_ from 08:00 to 18:00 (typical family household); ^(3)^ 6 W m^−2^_nfa_ 24/7 (typical senior household), results in 144 Wh m^−2^_nfa_ d^−1^, according to DIN 4108-2 ^(4)^ day and night ventilation, according to user model for manual window control. Abbreviations and symbols: H_T_ (mean heat transfer coefficient), ACH_min_ and ACH_max_ (minimum and maximum air change rate), S (solar heat gain factor), q_intern_ (daily internal heat gains), C (heat storage capacity), γ (offset temperature) and τ (time constant).

**Table 3:** Room temperatures from three monitored rooms in building m and simulation results from the minimum, mean, and maximum building model. The measured weather data are used in the simulation model. Here, the ambient air is shown for comparison only.

	Weather Ambient Air Temperature	Monitoring Room Temperature	Simulation Room Temperature	
overheating hours	230	243–325	169–611	h in 1 June–31 August
overheating degree hours	738	222–456	144–963	Kh in 1 June–31 August

## Data Availability

All recordings from the *DFG* project and the *DWD* weather data are publicly available. The *METRONA* measurement data have been previously published by F. Schröder, [27,44]. All raw data and the statistical analysis of all data are available on request from the author.
